# Calibration of self-report tools for physical activity research: the Physical Activity Questionnaire (PAQ)

**DOI:** 10.1186/1471-2458-14-461

**Published:** 2014-05-16

**Authors:** Pedro F Saint-Maurice, Gregory J Welk, Nicholas K Beyler, Roderick T Bartee, Kate A Heelan

**Affiliations:** 1 235 Forker Building, Ames, IA 50011, USA; 2 Mathematica Policy Research, Department of Statistics, 1100 1st Street, NE, Washington, DC 20002, USA; 3 Human Performance Lab, 1410 West 26th Street, Kearney, NE 68849, USA

**Keywords:** Youth, Accelerometry, Measurement, Public health

## Abstract

**Background:**

The utility of self-report measures of physical activity (PA) in youth can be greatly enhanced by calibrating self-report output against objectively measured PA data.

This study demonstrates the potential of calibrating self-report output against objectively measured physical activity (PA) in youth by using a commonly used self-report tool called the Physical Activity Questionnaire (PAQ).

**Methods:**

A total of 148 participants (grades 4 through 12) from 9 schools (during the 2009–2010 school year) wore an Actigraph accelerometer for 7 days and then completed the PAQ. Multiple linear regression modeling was used on 70% of the available sample to develop a calibration equation and this was cross validated on an independent sample of participants (30% of sample).

**Results:**

A calibration model with age, gender, and PAQ scores explained 40% of the variance in values for the percentage of time in moderate-to-vigorous PA (_%_MVPA) measured from the accelerometers (_%_MVPA = 14.56 - (sex*0.98) - (0.84*age) + (1.01*PAQ)). When tested on an independent, hold-out sample, the model estimated _%_MVPA values that were highly correlated with the recorded accelerometer values (r = .63) and there was no significant difference between the estimated and recorded activity values (mean diff. = 25.3 ± 18.1 min; p = .17).

**Conclusions:**

These results suggest that the calibrated PAQ may be a valid alternative tool to activity monitoring instruments for estimating _%_MVPA in groups of youth.

## Background

The development of more feasible and accurate methods of assessing physical activity behavior is an important public health research priority [1-4]. Objective monitoring devices have advantages but the high cost and burden of data processing make them impractical for large-scale applications [5-7]. Subjective (survey-based) tools are inexpensive and easy to use but they suffer from questionable validity [8]. Objective measures are often used to validate less accurate measures, such as subjective instruments, but this does not directly improve the accuracy or precision of the self-report instrument. If simple, easy-to-use self-report instruments could be calibrated against more accurate assessments, it might be possible to generate equivalent estimates of PA in a more efficient and cost-effective manner.

Calibration is a commonly accepted measurement practice that allows data to be scaled or adjusted to produce more accurate and usable estimates [9]. Considering the complexities of classifying and coding physical activity, it is actually quite naive to expect raw, uncalibrated, self-report estimates to even come close to individual-level estimates of PA [10]. However, questionnaires have been shown to be able to rank people according to their activity and estimate group-level PA in young populations with reasonable accuracy [6,10].

The use of measurement error models and calibration procedures are common in diet-related research so it is surprising that there are so few examples of measurement error studies [11] and calibration applications in studies of physical activity [12]. However, the growing interest in this topic is clear as illustrated by the conference on self-report measures, sponsored by the National Institutes of Health that highlighted the value of such measures and the need for continued refinement [13]. This topic has also been addressed in recent epidemiology research studies that demonstrated the importance of regression calibration for self-reported physical activity [14,15]. A number of other studies have also emphasized the importance of accurate self-report measures for epidemiology research, large school-based projects, and surveillance applications [14,16-18]. This paper helps address the need for more accurate self-report measures of youth by developing a robust calibration method for a commonly used physical activity self-report instrument in youth—the Physical Activity Questionnaire (PAQ)—which has been used to measure activity in children (PAQ-C) and adolescents (PAQ-A) [19,20].

The PAQ was selected because of its well-established psychometric properties and desirable measurement characteristics compared to other self-report measures for youth [21,22]. A review by Biddle and colleagues identified the PAQ as one of the most promising self-report tools available in the field [23]. Although the PAQ has shown good utility for field-based research [20,24-29], a limitation is that the outcome score is not readily interpretable [23]. The PAQ items are scored using ordinal scales (1–5 scale) and the outcome measure is computed as a simple mean of the individual items [30]. This makes it difficult to relate the PAQ score to established public health guidelines or to quantify levels of physical activity.

The purpose of this study was to develop and evaluate a calibration model that would allow raw PAQ scores to be converted to a more useful indicator of moderate-to-vigorous physical activity (MVPA) (namely, percentage of time in MVPA and/or minutes of MVPA) using an accelerometry-based activity monitor as the criterion measure. Accelerometers provide an objective indicator of free-living physical activity that can be temporally linked to data from a self-report tool [31]. Other “gold-standard” measures of physical activity (e .g. doubly labeled water, indirect calorimetry, and direct observation) cannot satisfy these objectives, and, therefore, are not well suited to this type of application.

## Methods

### Participants

The data for the study were collected as part of a school-based study to monitor activity that was conducted in fall 2009 and spring 2010. Participants (n = 261; 172 collected in the fall and 89 in the spring) were recruited from 12 schools (9 elementary and 3 secondary schools) from a small Midwestern community, Kearney, Nebraska, USA. Using a cluster sampling technique, 12 classrooms, grades 3 through 5, were randomly identified for sampling, along with 12 secondary-level classrooms. Participants were required to return both an assent and consent forms signed in order to participate in the study. The study was approved by the University of Nebraska Kearney Institutional Review Board.

### Instruments

#### Physical activity questionnaire

The PAQ, a self-administered 7-day (previous week) recall questionnaire, was designed to assess overall participation in PA. The PAQ-C was originally developed for use with elementary school children but was later adapted for middle school and high school youth (PAQ-A). The first item is an activity checklist that includes several common sports, leisure activities, and games. The developers of the PAQs said this item acts as an important memory cue, which might suggest it was not devised to get a precise indicator of activity [20]. The remaining items assess activity during specific periods of the day, including physical education (PE) class, recess (included only in the PAQ-C), lunch, immediately after school, evening, and the weekend, as well as two additional questions that assess overall activity patterns during the week. Each question is scored using a scale that ranges from 1 to 5; the higher score indicates a higher level of activity. The average of the items is used to create the final PAQ summary score. Previous studies have supported the validity of the PAQ instrument for assessing general levels of physical activity [20,24-29].

#### Actigraph GT1M

The ActiGraph GT1M (Actigraph, Pensacola, Florida, USA) activity monitor was selected as the criterion measure in the present study because of its wide acceptance and use in physical activity assessment research [32,33]. This activity monitor is a small, uniaxial accelerometer that is attached by a belt to the right side of the waist to capture acceleration movements from 0.05 to 2.0 g. It has a frequency band limit of 0.25-2.5 Hz. The GT1M uses a sampling rate of 30 Hz (meaning 30 measurements per second) and has 1 megabyte of memory. The available cut points for determining levels of MVPA were developed using an older version of Actigraph (CSA 7164), but those cut points can also be applicable to the GT1M as well [34].

### Procedures

Students who returned a completed informed consent and assent form were included in the study. Data on weight and height were obtained by standardized procedures and used to calculate body mass index (BMI). BMI percentiles were computed and described using Centers for Disease Control and Prevention growth charts (normal: BMI <85th percentile; overweight: BMI ≥85th and < 95th percentile; and obese: BMI ≥95th percentile). Upon collection of anthropometric information participants were asked to wear an ActiGraph accelerometer for 7 consecutive days, and instructed to remove the monitor only during water-based activities. The accelerometer was initialized to store activity counts every 30 seconds (i.e. for 30-second epochs).

After 7 days of wearing the accelerometer, participants were asked to return the monitor and complete a PAQ-C or a PAQ-A. Students in grades 3 through 5 completed a PAQ-C in their regular classroom while being supervised by the classroom teacher. Adolescents in secondary grades completed the PAQ-A during their PE class while being supervised by the PE teacher.

### Data processing

The average PAQ score (1–5 scale) was defined as the self-reported activity index using either the PAQ-C or the PAQ-A (including the recess item for children but not for adolescents). This score was computed using standard PAQ procedures as described by the developers of the questionnaires [30].

The Actigraph data were downloaded using the software provided by the manufacturer (version 5.0, Actigraph, Pensacola, Florida), and imported into SAS v9.2 for data processing and screening. Strict compliance criteria for the accelerometer were established to ensure appropriate calibration. A day was defined as extending from 8 a.m. to 9 p.m. to minimize the dilution of activity due to misclassification of awake time [35]. For a day to be deemed valid, participants had to have had ≥70% of valid data per day (equivalent to 9.0 hours a day); non-wear time was identified by continuous bouts of 90 minutes at 0 counts per minute allowing for 2 consecutive minutes of counts per minute from 0 to 100 [36,37]. There is no consensus on the most appropriate method to handle non-wear time however, we favored a longer non-wear bout criteria to account for extended periods of sitting (i.e., continuous bouts of counts equal to 0) that can occur during regular class time. For overall weekly PA levels to be counted, participants had to have at least 4 valid days of data (3 week days and 1 weekend day) [38]. Counts were converted to physical activity estimates using scaled (i.e., 30-seconds) age-specific cut points for the Actigraph [32]. A standard intensity definition for youth MVPA was used and set at ≥4 METS.

The average percentage of time spent in MVPA was computed as the number of minutes in MVPA divided by the number of minutes of wear time, and this was done separately for weekdays (_%_MVPA weekday) and weekend days (_%_MVPA weekend day).

### Statistical analyses

Data analyses were computed separately for the calibration and cross-validation samples. These two groups were randomly selected and defined to represent 70% (calibration; n = 103) and 30% (cross validation; n = 45) of the full sample. Separate one-way ANOVAs were done to examine differences between age groups and to evaluate differences between calibration and cross-validation groups. Differences in categorical outcomes were assessed using Pearson chi-square tests.

For the calibration analyses, the average percentage of time spent in MVPA (_%_MVPA) was defined as the dependent variable. This outcome measure was selected for the calibration phase (as opposed to MVPA_min_) because it is less likely to be influenced by sample-specific school schedules. For example, the number of MVPA minutes would be directly influenced by the frequency and duration of active periods during school time but the percentage of time in MVPA would not be influenced to the same degree. Another advantage of using _%_MVPA is that it can also minimize possible differences due to accelerometer wear time. The use of _%_MVPA is more abstract when compared to minutes of PA but it is expected to improve the external validity of the resulting PAQ calibration equation. Once the percentage estimates are determined, the weekly estimated minutes of MVPA can be easily computed by multiplying the predicted daily MVPA percentage by the total available minutes per week.

Multivariate linear regression was used to determine the relationship between the PAQ outcome score and the average daily percentage of time spent in MVPA (_%_MVPA) recorded by the Actigraph (calibration). Model fit was evaluated based on the model R^2^ values and Akaike Information Criterion (AIC) values [39], and the estimated β coefficients from each independent variable in the model, including the PAQ score. The root mean square error (RMSE) (also known as standard error of estimate (SEE) was used as an indicator of model accuracy and computed as the square root of the mean square residuals from the overall regression ANOVA table. Model precision was examined using the Breusch-Pagan test for heteroscedacity of residuals.

For cross validation, the model was applied to the remaining subsample of participants (30%) that were not included in the calibration analyses (an independent sample). Estimated daily _%_MVPA values were converted to weekly minutes of MVPA by multiplying the model-based _%_MVPA values by the total weekly minutes available for physical activity. For the present study, we assumed that youth would have approximately 13 hours a day to potentially be active (24 hours minus 11 hours of sleep/rest). Previous studies have reported average sleep time for children as high as 10.6 hours [35] so this is a reasonable approximation of available activity time. Thus, the _%_MVPA estimates were multiplied by a value of 5,460 minutes (13 hours × 7 days = 5,460 minutes) to obtain estimates of weekly MVPA minutes. This assumption may not be tenable for all youth but (as described above) the approach enabled us to produce estimates of MVPA that account for variability in activity time allocations during school time (e.g. recess duration and PE duration).

The validity of the calibration algorithm was first evaluated using a paired *t*-test to examine the overall difference between the two instruments and the proportion of variance explained (R^2^). The appropriateness of the developed calibration equation was discussed based on the RMSE (for group-level assessment) and limits of agreement (individual-level assessment) computed as the [mean difference ± (1.96 × standard deviation)] from the mean difference. Significance level was set at .05 and all analyses were conducted using SAS v9.2.

## Results

The final sample used for analysis (based upon an examination of participant compliance with the study protocol) included 148 youths (76 boys and 72 girls). The average wear time was 754.8 ± 24.7 minutes, which is close to the hypothesized 13 hours per day (780 minutes). Overall descriptive analyses were conducted for the full sample and separately for two different groups: calibration (n = 103) and cross-validation samples (n = 45). The two groups were similar in their demographic characteristics and each included a balanced sample of Fall and Spring observations in order to account for possible season differences in levels of PA.

Overall, students in the older age group were taller, heavier, and less active than their younger peers. These differences were consistent among the calibration and cross-validation samples, and they were supported by nonsignificant differences between the calibration and cross-validation in all variables (*p* > .05) except in PAQ scores [*F* (1,146) = 4.13, *p* = .04)] (Table 1).

**Table 1:** Descriptives for the calibration and cross-validation samples stratified by age group

**Age group**	**Younger**	**Older**	**Combined**	
***Calibration sample***	**n = 71**	**n = 32**	**n = 103**	
	**M ± SD**	**M ± SD**	**M ± SD**	**p**
Age (y)	9.7 ± 1.1	13.3 ± 1.3	10.8 ± 2.0	<.001
Gender (% male)^1^	47.9	46.9	47.6	.920
Height (cm)	141.0 ± 7.9	160.5 ± 11.7	146.8 ± 12.7	<.001
Weight (kg)	37.4 ± 9.8	60.7 ± 19.3	44.6 ± 17.2	<.001
BMI (kg/m^2^)	18.6 ± 3.8	23.2 ± 5.6	20.1 ± 4.9	<.001
Obese (%)^1^	12.7	28.1	17.4	.056
Spring (%)^1^	57.8	56.2	57.3	.800
MVPA_min_ (min)	60.7 ± 23.1	37.6 ± 20.1	53.5 ± 24.6	<.001
MVPA weekday (%)	8.0 ± 3.3	5.0 ± 2.5	7.1 ± 3.3	<.001
MVPA weekend (%)	8.0 ± 4.2	4.9 ± 5.3	7.0 ± 4.8	<.001
AA (cpm/day)	475.4 ± 114.7	429.8 ± 137.9	461.2 ± 123.5	.080
AM wear time (d)	6.0 ± 0.9	5.6 ± 0.9	5.9 ± 0.9	.025
PAQ (0–5 scale)	3.2 ± 0.7	2.8 ± 0.6	3.1 ± 0.7*	.005
***Cross-validation sample***	**n = 32**	**n = 13**	**n = 45**	
	**M ± SD**	**M ± SD**	**M ± SD**	**p**
Age (y)	8.4 ± 3.1	13.1 ± 1.0	10.6 ± 1.9	<.001
Gender (% male)^1^	62.5	53.9	60	.590
Height (cm)	140.7 ± 8.4	157.0 ± 10.9	145.3 ± 11.7	<.001
Weight (kg)	37.3 ± 9.4	46.9 ± 11.0	40.1 ± 10.7	.001
BMI (kg/m^2^)	18.7 ± 3.6	18.9 ± 2.8	18.8 ± 3.3	.890
Obese (%)^1^	0	0	0	na
Spring (%)^1^	46.9	61.5	51.1	.370
MVPA_min_ (min)	61.4 ± 21.7	39.0 ± 19.4	55.7 ± 22.2	<.001
MVPA week day (%)	8.4 ± 3.1	5.5 ± 2.6	7.6 ± 3.2	.005
MVPA weekend day (%)	8.4 ± 4.7	4.0 ± 3.0	7.1 ± 4.7	.003
AA (cpm/day)	491.0 ± 100.5	434.6 ± 125.1	474.7 ± 109.8	.120
AM wear time (d)	6.2 ± 0.8	5.5 ± 1.1	6.0 ± 0.9	.014
PAQ (0–5 scale)	3.5 ± 0.5	2.9 ± 0.5	3.3 ± 0.6	<.001

p-values relate to age group comparison tests.

^1^Tested using Pearson chi-square test.

*Significantly different than the cross-validation sample.

M ± SD = mean ± standard deviation.

na = not applicable.

AA = Average activity.

AM = Activity monitor.

### Calibration

A multivariate linear regression model was fit to the calibration sample with three independent variables: PAQ score, age (in years; no decimal places), and gender (boys = 1; girls = 2). The _%_MVPA was defined as the dependent variable. BMI was not considered because it might not be feasible to obtain BMI scores when computing youth activity levels from large samples (especially in school settings). Nevertheless, the utility of BMI was examined and deemed to be nonsignificant (*p* = .26) when included in the calibration model.

**Figure 1:**
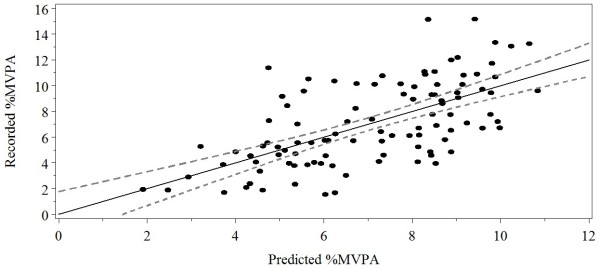
**Relationship between accelerometer activity levels (recorded **_**%**_**MVPA) and predicted activity levels (predicted **_**%**_**MVPA) in the calibration sample.** The solid line represents the best fit with the respective 95% confidence interval for the mean predicted values (dashed lines).

Examination of Spearman (for PAQ) correlation, revealed moderate and significant linear associations of _%_MVPA with PAQ scores (*r*_*s*_(103) = .35, *p* < .001). This supported the inclusion of this variable in the model and justified the decision to proceed with linear forms of the main independent variable. The final model explained 40% of the variability in _%_MVPA [(*R*^*2*^ = .40; *F* (3,99) = 22.10, *p* < .001)], and the estimated β coefficients for age (β = −0.84 ± 0.13; *p* < .001) and PAQ (β = 1.01 ± 0.39; *p* = .01) variables were found to be significant predictors of _%_MVPA. Gender approached significance (β = −0.98 ± 0.51; *p* = .06), and was retained in the model to account for possible population differences between boys’ and girls’ activity (Figure 1).

AIC values were computed for this model fit (with PAQ, age, and gender predictors) and other models with additional variables (namely BMI and interactions between BMI, PAQ, age, and gender). The AIC value for the simple model was similar to AIC values for more complex models, suggesting that the simple model with PAQ, age, and gender predictors is reasonable. The final model for estimation of (daily) _%_MVPA was as follows:

$$\begin{array}{l} \mathrm{Dail}y_{\%}\mathrm{MVPA}=14.56‒\left(\mathrm{sex}*0.98\right)‒\left(0.84*\mathrm{age}\right)\\ \;+\left(1.01*\mathrm{PAQ}\right) \end{array}$$

Sex was coded as “1” if male and “2” if female; age was coded in years (ranging from 8 y to 14y); PAQ was the average raw score with one decimal place.

The overall accuracy of the model was equal to 2.54% (RMSE = 2.54%) and indicated a reasonable fit to the data (suggesting that the equation could estimate group-level _%_MVPA with an error of 2.54%). The Breusch-Pagan test (a test for heteroscedacity) showed that the error variability (precision) was consistent across different levels of accelerometer activity (*X*^*2*^ (8) = 10.7; *p* = .22). Calibration regression coefficients are presented in Table 2.

**Table 2:** Calibration parameters and model evaluation indices

	**Estimate**	**SE**	**T**	**p**
***Model parameters***				
Intercept	14.56	2.14	6.81	<.001
Gender	−0.98	0.51	−1.93	.06
Age	−0.84	0.13	−6.74	<.001
PAQ	1.01	0.39	2.58	.01
***Model evaluation***				
R^2^	0.4			
RMSE	2.54			
VIF^a^	1.02-1.05			
Breusch-Pagan	10.7			.22

SE = standard error.

RMSE = root mean square error.

^a^VIF = variance inflation factor (range).

Dependent variable = _%_MVPA (average per day).

Gender: boys = 1; girls = 2.

Age: in years (e.g. 10).

### Cross validation

**Figure 2:**
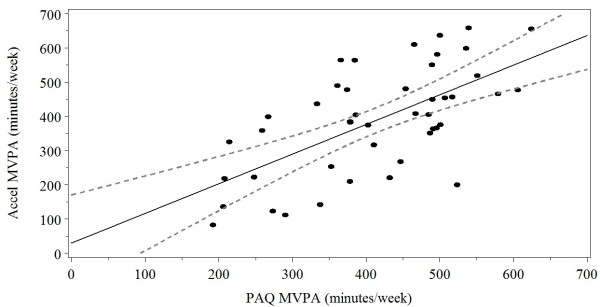
**Relation between predicted and recorded minutes of MVPA in the cross-validation sample.** The solid line represents the best fit line and the dashed lines represent the 95% confidence bounds about the best fit line.

Estimates of daily percentage of time in MVPA from the calibration model (_%_MVPA) were multiplied by 5,460 minutes of weekly awake time to estimate total weekly minutes of MVPA in the cross-validation sample (n = 45). Model-estimated values were compared to observed accelerometer-based values of MVPA. On average, the PAQ calibration equation produced similar accelerometer estimates of total minutes of MVPA (Mean _diff._ = 25.3 ± 18.1 minutes (*t* (44) = 1.40, *p* = .17). Accelerometer and model-estimated minutes of weekly MVPA were moderately and significantly associated with each other, and the estimated scores explained 40% of the variability in accelerometer minutes of weekly MVPA with an RMSE of 121.6 (*R*^*2*^ = .40, *F*(1,43) = 28.71, *p* < .001) (Figure 2).

**Figure 3:**
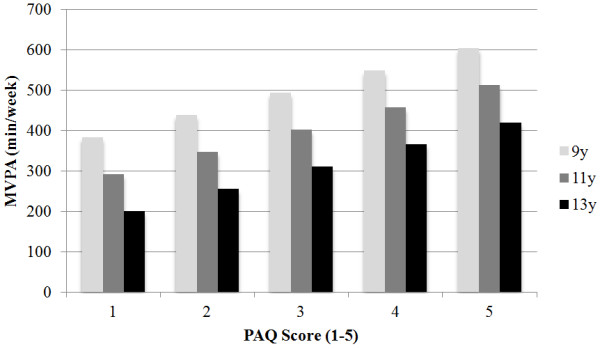
**Predicted minutes of MVPA (min/week) using different PAQ scores.** Estimates were generated for three boys aged 9, 11, and 13 years. The final estimated score was divided by 100 and multiplied by 5,460 minutes as a measure of weekly activity. For each PAQ score unit increase there was an increase of 55.1 minutes of weekly MVPA.

The Pearson correlation between absolute error and accelerometer estimates of MVPA was equal to -.24 (*p* = .11), supporting the assumption of homogenous distribution of error. Limits of agreement (LOA) were determined to examine individual and group-level error. The 95% confidence interval for group-level bias suggested that this error can range from between −6% and 16% of accelerometer group estimates (deemed nonsignificant). Individual error ranged from between −56% and +69% of the accelerometer value (Table 3).Figure 3 provides an illustration of the relationship between PAQ scores and estimated minutes of MVPA (min/week). Results are described for boys aged 9, 11, and 13 years. Each unit increase in the final PAQ score (1–5 scale) was associated with an increase of 55.1 minutes of weekly MVPA.

**Table 3:** Agreement between weekly minutes of MVPA obtained from the PAQ and accelerometer

	**PAQ MVPA**^**1**^	**Acc MVPA**^**1**^	**Mean Bias**^**1**^	**95% CI**^**2**^	**LOA**^**3**^
**Estimate**	415.2 ± 113.3	389.9 ± 155.3	25.3 ± 121.2	−11.2, 61.7	−212.3, 262.9

^1^Mean ± standard deviation.

^2^95% confidence interval for the average mean difference (PAQ - Acc).

^3^Limits of Agreement computed as: mean difference ± (1.96 *x standard deviation of mean difference).

## Discussion

Self-report instruments have been used in many epidemiology studies and have contributed to most of what is known regarding the relationship between physical activity and health [40]. Although objective instruments are now widely used, there is considerable need to improve the utility and accuracy of self-report measures. The low cost and ease of use make self-report measures the most feasible approach for assessing physical activity profiles in large and diverse groups of individuals [40]. The calibration procedures tested in this study provide a way to scale self-report data so it matches data obtained using objective measures. In this study, we calibrated the PAQ (a widely used self-report instrument in research with children) but the methods would have similar utility for other self-report instruments. The specific goal was to evaluate the validity of a simple calibration equation to convert raw PAQ scores into a more meaningful outcome measure (minutes of MVPA per week) and determine if they can be used to predict group-level estimates of MVPA.

The results support the utility of this method. The resulting model estimated objectively recorded activity with an error of 2.54%, and it explained 40% of the variability in MVPA. A strength of the analytic approach is that the calibration equation was developed to predict the percentage of time spent in MVPA across the week. This value is then converted to minutes of MVPA by multiplying by total weekly minutes considered in this study (5,460 minutes of awake time, or 8 a.m. to 9 p.m., Monday through Sunday). This approach is more robust than directly estimating minutes of MVPA because it avoids potential error caused by future differences in the length of the typical day being considered (external validity). The approach also minimizes any wear time differences between participants.

The utility of this approach was demonstrated in the cross-validation analyses as reasonable measurement agreement was obtained when it was evaluated in an independent sample. The 95% confidence interval for group mean differences indicated that group-level bias can range from −11.2 to 61.7 minutes of MVPA, equivalent to −6% and 16% of accelerometer estimates of weekly minutes of MVPA. This supports the ability of the PAQ algorithm to estimate group-level estimates of accelerometer activity. The results from this independent sample are noteworthy because they demonstrate that the calibration algorithm is effective in estimating activity in a different group of individuals. Although the results are promising, there is clearly significant room for improvement in the accuracy of this type of calibration.

As stated, a potential application for this type of calibrated tool would be to use it in place of more expensive and cumbersome objective monitors. To evaluate the potential utility for this type of application, we retrospectively identified youth who would meet public health guidelines (e.g. 60 minutes of daily MVPA) [41] based on both the PAQ and accelerometer data. These results revealed a moderate and significant degree of agreement (a*rea under the curve* = 0.79 ± 0.07, *p* < .001). Approximately 65% of non-active individuals based on the accelerometer data were correctly identified through self-reported estimates (specificity = 65.4). Approximately 74% of active individuals meeting guidelines with the accelerometer were correctly identified by the PAQ (sensitivity = 73.7). These results are reasonable, considering the large discrepancies that have previously been reported between self-report and objective measures in past epidemiology studies. For example, Troiano et al. (2008) examined accelerometer data from the NHANES 2003–2004 cohort and found that only 2.3% to 3.5% of adults met the physical activity guidelines for Americans (PAGA) [36]. Similarly, Tucker and colleagues [42] found that the prevalence of adults meeting the PAGA, based on accelerometry, was 9.6%, even as the estimate was 62.0% when activity was self-reported. The prevalence rates of adults meeting the PAGA reported in these two studies based on accelerometer data were substantially lower than PAGA compliance based on self-reported activity. These discrepancies with self-report data have been well chronicled, but with calibration approaches similar to those demonstrated here it would be possible to model self-report data so they approximate the patterns and distribution from objective data.

The approach presented here would provide reasonably accurate group-level estimates. It is important to note, however, that we observed large individual bias ranging from −212.3 and 262.9 minutes or −56% to +69% of the accelerometer estimates of MVPA. Thus, it may be premature to apply the equation for individual estimation. However, this issue is not unique for our application. Current calibration equations used to process and summarize accelerometer data have also been shown to have limitations for estimating individual data.

The results of the present study support the value and potential of this calibration approach, but it is important to consider the inherent differences between objective (e.g. Actigraph monitor) and subjective measures (e.g. self-report) of physical activity since it may directly explain some of the findings. Accelerometry-based activity monitors (e.g. Actigraph) provide direct measures of limb acceleration and output raw counts accumulated per pre-defined unit of time. Subjective, self-report measures, on the other hand, provide contextual information about PA behaviors that may not be associated with acceleration of the limbs. Both instruments are essentially measuring different aspects of the same underlying behavior. Based on this, it is actually quite naive to expect that these two instruments can provide equivalent information. The advantage of calibration procedures demonstrated in this paper is that it is possible to establish quantitative links between the subjective reports and more objectively monitored data. The methodology has clear promise but refinements will be needed to enable more accurate estimates at the individual level.

Aspects of the design and the nature of the measures may have limited our ability to fully calibrate the PAQ. The For example, the accelerometer data were collected using 30-second epochs and this may have obscured shorter and more intermittent bouts of activity. However, this limitation is somewhat minimized when activity is aggregated into MVPA, as in the present study [43]. Also relevant was the fact that the current calibration utilized data collected across a full week rather than treating weekdays and weekends separately. It may be possible to create more effective calibration equations by directly matching the reported times with the data recorded from the accelerometer. This was not possible in the present analyses because the purpose of the study was to calibrate the original PAQ as recommended by the developers. It is noteworthy that the present calibration equation yielded reasonable group-level estimates despite these limitations. Nevertheless, the equation should be used with caution until more robust evaluations are performed. The developed equation, for example, should be tested on another group of individuals across different age groups. Despite the randomized distribution of participants into calibration and cross-validation groups, no obese children were included in the cross-validation sample. The majority of our sample was composed of individuals 8 to 13 years old, and, therefore, the results should not generalize to older individuals.

## Conclusions

The results demonstrate that the PAQ can be calibrated to provide accurate group-level estimates of MVPA. The findings presented here are specific to the PAQ, but similar approaches can be used to improve the utility of other self-report instruments. There is clear public health interest in improving self-report measures [13], and the calibration procedures shown here offer a way to get reasonable accuracy with a more feasible and cost-effective strategy.

## Competing interests

The following authors declared no competing interests: PSM, GW, NKB, TB, KH.

## Authors’ contributions

PSM performed the statistical analysis and drafted the manuscript. GW participated in the design of the study and drafted the manuscript. NKB participated in the statistical analysis and helped drafting the manuscript. TB participated in the design of the study and coordination and helped drafted the manuscript. KH conceived of the study, and was responsible for its design and coordination and helped drafted a manuscript. All authors approved the final manuscript.

## Pre-publication history

The pre-publication history for this paper can be accessed here:

http://www.biomedcentral.com/1471-2458/14/461/prepub

## References

[B1] Bauman A Phongsavan P Schoeppe S Owen N Physical activity measurement - a primer for health promotion Promot Educ 2006 13 92 103 10.1177/10253823060130020103 17017286

[B2] Troiano RP A timely meeting: objective measurement of physical activity Med Sci Sports Exerc 2005 37 S487 S489 10.1249/01.mss.0000185473.32846.c3 16294111

[B3] Welk G Welk G Introduction to Physical Activity Research Physical Activity Assessment for Health-Related Research 2002 Champaign, IL: Human Kinetics 3 18

[B4] Wanner M Probst-Hensch N Kriemler S Meier F Bauman A Martin BW What physical activity surveillance needs: validity of a single-item questionnaire Br J Sports Med 2013 0 1 7 Published Online First June 14 10.1136/bjsports-2012-092122 23770662

[B5] Rennie KL Wareham NJ The validation of physical activity instruments for measuring energy expenditures: problems and pitfalls Public Health Nutr 1998 1 265 271 10933427 10.1079/phn19980043

[B6] Warren JM Ekelund U Besson H Mezzani A Geladas N Vanhees L Assessment of physical activity - a review of methodologies with reference to epidemiological research: a report of the exercise physiology section of the European Association of Cardiovascular Prevention and Rehabilitation Eur J Cardiovasc Prev Rehabil 2010 17 127 139 10.1097/HJR.0b013e32832ed875 20215971

[B7] Shephard RJ Limits to the measurement of habitual physical activity by questionnaires Br J Sports Med 2003 37 197 206 discussion 206 10.1136/bjsm.37.3.197 12782543 PMC1724653

[B8] Helmerhorst HJ Brage S Warren J Besson H Ekelund U A systematic review of reliability and objective criterion-related validity of physical activity questionnaires Int J Behav Nutr Phys Act 2012 9 103 10.1186/1479-5868-9-103 22938557 PMC3492158

[B9] Carroll R Ruppert D Stefanski L Crainiceanu C Regression Calibration Measurement Error in Nonlinear Models: A Modern Perspective 2006 2 Boca Raton, FL, U.S: Taylor and Francis Group 65 95

[B10] Corder K van Sluijs EM Wright A Whincup P Wareham NJ Ekelund U Is it possible to assess free-living physical activity and energy expenditure in young people by self-report? Am J Clin Nutr 2009 89 862 870 10.3945/ajcn.2008.26739 19144732

[B11] Nusser SM Beyler NK Welk GJ Carriquiry AL Fuller WA King BM Modeling errors in physical activity recall data J Phys Act Health 2012 9 Suppl 1 S56 S67 22287449 10.1123/jpah.9.s1.s56

[B12] Tucker JM Welk G Nusser SM Beyler NK Dzewaltowski D Estimating minutes of physical activity from the previous day physical activity recall: validation of a prediction equation J Phys Act Health 2011 8 71 78 21297187 10.1123/jpah.8.1.71

[B13] Bowles HR Measurement of active and sedentary behaviors: closing the gaps in self-report methods J Phys Act Health 2012 9 Suppl 1 S1 S4 22287442 10.1123/jpah.9.s1.s1

[B14] Tooze JA Troiano RP Carroll RJ Moshfegh AJ Freedman LS A measurement error model for physical activity level as measured by a questionnaire with application to the 1999–2006 NHANES questionnaire Am J Epidemiol 2013 177 1199 1208 10.1093/aje/kws379 23595007 PMC3664335

[B15] Neuhouser ML Di C Tinker LF Thomson C Sternfeld B Mossavar-Rahmani Y Stefanick ML Sims S Curb JD Lamonte M Seguin R Johnson KC Prentice RL Physical activity assessment: biomarkers and self-report of activity-related energy expenditure in the WHI Am J Epidemiol 2013 177 576 585 10.1093/aje/kws269 23436896 PMC3626043

[B16] Hallal PC Andersen LB Bull FC Guthold R Haskell W Ekelund U Global physical activity levels: surveillance progress, pitfalls, and prospects Lancet 2012 380 247 257 10.1016/S0140-6736(12)60646-1 22818937

[B17] Bull FC Maslin TS Armstrong T Global physical activity questionnaire (GPAQ): nine country reliability and validity study J Phys Act Health 2009 6 790 804 20101923 10.1123/jpah.6.6.790

[B18] Dumith SC Hallal PC Reis RS Kohl HW 3rd Worldwide prevalence of physical inactivity and its association with human development index in 76 countries Prev Med 2011 53 24 28 10.1016/j.ypmed.2011.02.017 21371494

[B19] Bailey DA The Saskatchewan pediatric bone mineral accrual study: bone mineral acquisition during the growing years Int J Sports Med 1997 18 Suppl 3 S191 S194 9272847 10.1055/s-2007-972713

[B20] Crocker PR Bailey DA Faulkner RA Kowalski KC McGrath R Measuring general levels of physical activity: preliminary evidence for the physical activity questionnaire for older children Med Sci Sports Exerc 1997 29 1344 1349 10.1097/00005768-199710000-00011 9346166

[B21] Chinapaw MJ Mokkink LB van Poppel MN van Mechelen W Terwee CB Physical activity questionnaires for youth: a systematic review of measurement properties Sports Med 2010 40 539 563 10.2165/11530770-000000000-00000 20545380

[B22] Tessier S Vuillemin A Briancon S Revue des questionnaires de mesure de l’activite physique valides chez les enfants et les adolescents Sci Sports 2007 43 3 118 125

[B23] Biddle SJ Gorely T Pearson N Bull FC An assessment of self-reported physical activity instruments in young people for population surveillance: Project ALPHA Int J Behav Nutr Phys Act 2011 8 1 10.1186/1479-5868-8-1 21194492 PMC3019119

[B24] Kowalski C Crocker PRE Kowalski NP Convergent Validity of the Physical Activity Questionnaire for Adolescents Pediatr Exerc Sci 1997 9 342 352

[B25] Kowalski K Crocker PRE Faulkner RA Validation of the physical activity questionnaire for older children Pediatr Exerc Sci 1997 9 174 186

[B26] Crocker PR Eklund RC Kowalski KC Children’s physical activity and physical self-perceptions J Sports Sci 2000 18 383 394 10.1080/02640410050074313 10902673

[B27] Moore JB Hanes JC Jr Barbeau P Gutin B Trevino RP Yin Z Validation of the physical activity questionnaire for older children in children of different races Pediatr Exerc Sci 2007 19 6 19 17554153 10.1123/pes.19.1.6

[B28] Janz KF Lutuchy EM Wenthe P Levy SM Measuring activity in children and adolescents using self-report: PAQ-C and PAQ-A Med Sci Sports Exerc 2008 40 767 772 10.1249/MSS.0b013e3181620ed1 18317366

[B29] Martínez-Gómez D Martínez-de-Haro V Pozo T Welk GJ Villagra A Calle ME Marcos A Veiga OL Reliability and validity of the PAQ-A questionnaire to assess physical activity in Spanish adolescents Rev Esp Salud Publica 2009 83 427 439 10.1590/S1135-57272009000300008 19701574

[B30] Kowalski K Crocker PRE Donen RM The Physical Activity Questionnaire for Older Children (PAQ-C) and Adolescents (PAQ-A) manual Book The Physical Activity Questionnaire for Older Children (PAQ-C) and Adolescents (PAQ-A) manual 2004 City: University of Saskatchewan: Saskatoon

[B31] Sirard JR Pate RR Physical activity assessment in children and adolescents Sports Med 2001 31 439 454 10.2165/00007256-200131060-00004 11394563

[B32] Freedson P Pober D Janz KF Calibration of accelerometer output for children Med Sci Sports Exerc 2005 37 S523 S530 10.1249/01.mss.0000185658.28284.ba 16294115

[B33] De Vries SI Van Hirtum HW Bakker I Hopman-Rock M Hirasing RA Van Mechelen W Validity and reproducibility of motion sensors in youth: a systematic update Med Sci Sports Exerc 2009 41 818 827 10.1249/MSS.0b013e31818e5819 19276851

[B34] Kozey SL Staudenmayer JW Troiano RP Freedson PS Comparison of the ActiGraph 7164 and the ActiGraph GT1M during self-paced locomotion Med Sci Sports Exerc 2010 42 971 976 10.1249/MSS.0b013e3181c29e90 19997000 PMC2893387

[B35] Esliger DW Copeland JL Barnes JD Tremblay MS Standardizing and optimizing the use of accelerometer data for free-living physical activity monitoring J Phys Act Health 2005 3 366 383

[B36] Troiano RP Berrigan D Dodd KW Masse LC Tilert T McDowell M Physical activity in the United States measured by accelerometer Med Sci Sports Exerc 2008 40 181 188 10.1249/mss.0b013e31815a51b3 18091006

[B37] Choi L Liu Z Matthews CE Buchowski MS Validation of accelerometer wear and nonwear time classification algorithm Med Sci Sports Exerc 2011 43 357 364 20581716 10.1249/MSS.0b013e3181ed61a3 PMC3184184

[B38] Trost SG Pate RR Freedson PS Sallis JF Taylor WC Using objective physical activity measures with youth: how many days of monitoring are needed? Med Sci Sports Exerc 2000 32 426 431 10.1097/00005768-200002000-00025 10694127

[B39] Akaike H A new look at the statistical model identification IEEE Trans Autom Control 1974 19 716 723 10.1109/TAC.1974.1100705

[B40] Haskell WL Physical activity by self-report: a brief history and future issues J Phys Act Health 2012 9 Suppl 1 S5 S10 22287448 10.1123/jpah.9.s1.s5

[B41] PAGAC Physical Activity Guidelines Advisory Committee Report, 2008 Book Physical Activity Guidelines Advisory Committee Report, 2008 2008 City: Washington, DC: U.S: Department of Health and Human Services

[B42] Tucker JM Welk GJ Beyler NK Physical activity in U.S.: adults compliance with the physical activity guidelines for Americans Am J Prev Med 2011 40 454 461 10.1016/j.amepre.2010.12.016 21406280

[B43] Nilsson A Ekelund U Yngve A Sjostrom M Assesing physical activity among children with accelerometers using different time sampling intervals and placements Pediatr Exerc Sci 2002 14 87 96

